# A systematic review of post-stroke fatigue measurement scale based on COSMIN guidelines

**DOI:** 10.3389/fneur.2024.1411472

**Published:** 2024-09-10

**Authors:** Lingsha Wu, Haiqin Jin

**Affiliations:** The Second Hospital of Jiaxing, Jiaxing, China

**Keywords:** post-stroke fatigue, measurement scales, COSMIN guide, systematic review, fatigue severity scale

## Abstract

**Objective:**

This study aimed to evaluate the methodological quality and measurement attribute quality of the post-stroke fatigue measurement scale, so as to provide some basis for the clinical application and promotion of related scales.

**Methods:**

The Chinese National Knowledge Infrastructure, the Wanfang Data Knowledge Service Platform, the China Science and Technology Journal Database, the Chinese Medical Journal Full-text Database, the Chinese Biology Medicine, PubMed, Embase, Medline, the Cochrane Library, the Web of Science, CINAHL, and PsycINFO databases were searched for literature on the post-stroke fatigue measurement scale up to June 2022. Literature screening and data extraction were carried out independently by two researchers, and in the case of disagreement, discussions were held with a third investigator to reach an agreement, and the COSMIN checklist and criteria were used to systematically evaluate the attributes of the measurement scale.

**Results:**

A total of 17 studies were included, involving 10 post-stroke fatigue measurement scales. The content validity of FSS-7, FACIT-F, NRS-FRS, and MFI-20 was “not mentioned,” and the remaining scales were “uncertain.” In terms of construct validity, MFS was “adequate”; FSS-7, FACIT-F, and NRS-FRS were “not mentioned”; and the remaining scales were “uncertain.” In terms of internal consistency, NRS-FRS was “not mentioned”; FSS and MFS were “adequate”; and the remaining scales were “uncertain.” In terms of hypothesis testing, CIS and FACIT-F were “not mentioned,” NRS-FRS was “adequate,” and the remaining scales were “uncertain.” The stability of FSS-7, CIS, FACIT-F, and MFI-20 was “not mentioned,” and the remaining scales were “adequate.” The cross-cultural validity of FSS-7 was “adequate,” and the remaining scales were “not mentioned.” All 10 scales were given a recommendation grade of “B”.

**Conclusion:**

For the time being, the FSS can be recommended to measure post-stroke fatigue, but it still needs to be tested for more relevant measurement properties in order to gain more support from high-quality evidence. For a more comprehensive assessment of post-stroke fatigue, the FIS, FAS, and NFI-stroke should perhaps be considered, as the FSS is a one-dimensional scale that can only measure physical fatigue in patients; however, these scales also need to be tested for more relevant measurement properties to verify their clinical applicability.

## 1 Introduction

Post-stroke fatigue is not related to tension and is a subjective feeling of stroke survivors about weakness and tiredness (1, 2). PSF arises not only from physical activities but also from mental or social activities. As one of the common complications after stroke, it has a high incidence, which will make it difficult or impossible for patients to maintain daily activities, thus causing a certain degree of adverse effects on their quality of life (3, 4). Accurate measurement of PSF is the premise and basis for the timely and effective treatment of the disease. There are many scales used to measure PSF, such as the fatigue severity scale (FSS) (5), fatigue impact scale (FIS) (6), and fatigue assessment scale (FAS) (7). Kjeverud et al. (8) explored the frequency and overlap of PSF by using scales, such as FSS, and the results showed that different scales produced different results. Blackwell et al. (9) noted that there are currently no corresponding guidelines to assess fatigue management in patients with PSF in fatigue management and that there are no established guidelines yet. Thus it can be seen different measurement focus of different scales, it has not been able to determine whether these scales have good measurement properties, and few studies to systematically evaluate these measurement properties. The guidelines for the selection criteria for health measurement tools (COSMIN) (10) can assess the methodological quality and measurement attribute quality of the scale, and the best scale for the purpose of the study can be selected. In this study, a systematic evaluation of PSF measurement scales using COSMIN quality standards was carried out to clarify the methodological quality and measurement attribute quality of relevant scales. It aimed to comprehensively evaluate the evidence level of each measurement attribute, leading to the final recommendation and providing certain evidence-based support for the application and promotion of relevant scales in clinical practice.

## 2 Materials and methods

### 2.1 Inclusion criteria and exclusion criteria

Inclusion criteria were as follows: ① study subjects were stroke patients; ② the study includes the measurement performance evaluation of the PSF measurement scale; ③ at least one measurement attribute was evaluated on the scale; ④ access to the full text of the Chinese and English literature, where nationality is not limited. Exclusion criteria were as follows: ① review, systematic evaluation, conference, animal experiments, qualitative research, cases, and other types of literature; ② the evaluation tool is only used to study the current status of its application and collect research subject data or the literature measuring outcome indicators.

### 2.2 Literature retrieval strategy

Literature on PSF measurement published from the database until June 2022 in the Chinese Journal Full-text Database, the Wanfang Data Knowledge Service Platform, the VIP Database, the Chinese Medical Journal Full-text Database, the Chinese Biomedical Literature Database, PubMed, Embase, Medline, the Cochrane Library, the Web of Science, CINAHL, and PsycINFO databases were being retrieved using a computer. The literature search was completed by the combination of subject words and free words, and the gray literature search was performed.

### 2.3 Literature screening and data extraction

Two researchers who had participated in the relevant training and fully mastered the COSMIN evaluation criteria independently completed the literature screening and data extraction according to the inclusion and exclusion criteria and cross-checked the results. Once a disagreement occurs, they discuss it with the third investigator to reach a consensus. The contents of data extraction include those as follows: the first author, year of publication, country, scale name, sample size, scale dimension, scoring method used for each item, scale evaluation time, and retest time.

### 2.4 Evaluation steps

Two investigators independently completed the quality of PSF, the quality of measurement attributes, and the level of evidence using the COSMIN risk of bias tool (10) and performed a cross-check. In the case of disagreement, they discussed it with the third investigator to reach an agreement. The contents of data extraction include those as follows: the first author, year of publication, country, scale name, sample size, scale dimension, scoring method used for each item, scale evaluation time, and retest time.

### 2.5 Study tools

#### 2.5.1 Methodological quality evaluation

The methodological quality of the included scale was assessed according to the COSMIN risk of bias checklist (11). A total of 10 modules need to be evaluated, namely, content validity scale development, content validity, structure validity, internal consistency, cross-cultural validity or measurement invariance, stability, measurement error, validity and criterion validity, hypothesis testing, and responsiveness. The risk of bias of each item in the module was evaluated with the result of “very good” “adequate” or “doubtful” or “inadequate,” and then the minimum evaluation of all the entries in a module was taken as the total evaluation result of the module.

#### 2.5.2 Quality evaluation of the measurement properties

The quality of the nine measurement attributes of content validity, construct validity, internal consistency, stability, measurement error, hypothesis testing, cross-cultural validity or measurement invariance, criterion validity, and responsiveness were evaluated according to the COSMIN quality specification (12), and the evaluation rating is “sufficient (+)” or “inadequate (−)” or “uncertain (?).” When a measurement attribute of a scale is “sufficient (+)” or “inadequate (−)” or “uncertain (?),” the overall rating of this measurement attribute is also “full (+)” or “inadequate (−) “or “unsure (?).” In the meantime, when a measurement attribute of the scale is not evaluated consistently among studies, and the reason cannot be explained, then the overall rating of the measurement attribute is also “inconsistent (±).”

#### 2.5.3 Evaluation of the evidence grade

The inclusion scale was assessed according to GRADE (13), evaluating it based on the risk of bias, inconsistency, imprecision, and indirectness. COSMIN first identified the measurement attributes of the measurement scale as “high quality,” then downgraded according to the above four aspects, and divided the level of evidence into “high” or “medium” or “low” or “extremely low.” Subsequently, opinions on the recommended strength of the scale were formed based on the evidence evaluation results. The recommended strength of the scale is “A” or “B” or “C”; “A” is recommended, “C” is not recommended, and “B” is between “A” and “C,” indicating that the scale has some potential, but more studies need to be conducted to verify its effectiveness. The content validity of the recommended strength “Grade A” is “sufficient” and the internal consistency level is not “low”; “Grade C” is proven insufficient; and “Grade B” corresponds to neither “Grade A” nor “Grade C”

## 3 Results

### 3.1 Literature search results

**Figure 1:**
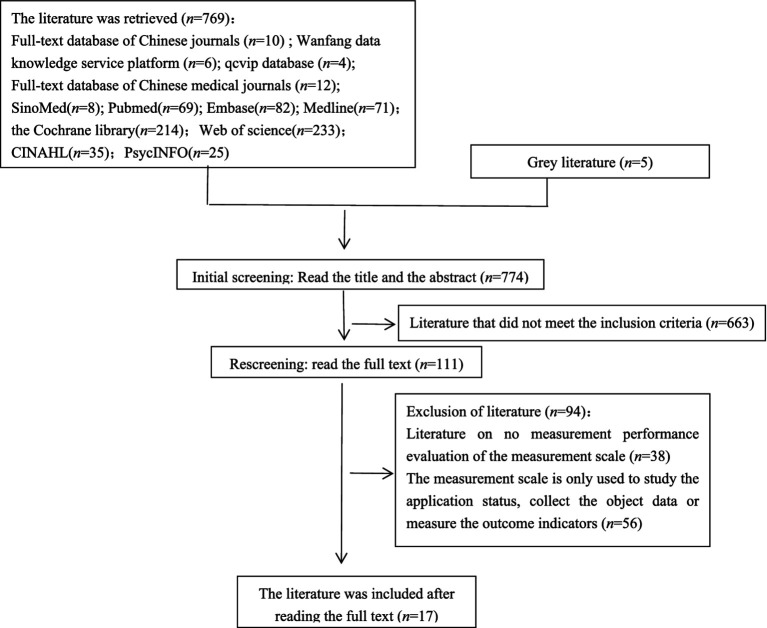
Flow chart of literature screening.

In total, 774 articles were screened according to inclusion and exclusion criteria, and 17 articles were finally included. The literature screening process is shown in Figure 1.

### 3.2 Basic characteristics of the included literature

A total of 17 literature articles were included in this study (5–7, 14–27), involving 10 PSF-related measurement scales, namely, the Fatigue Severity Scale (FSS), Fatigue Severity Scale (7 entries) (FSS-7), Fatigue Impact Scale (FIS), Fatigue Assessment Scale (FAS), Stroke nerve fatigue Index Scale (NFI-Stroke), Personal Fatigue Strength Questionnaire (CIS), Functional Assessment of Chronic Disease Treatment-Fatigue Scale (FACIT-F), Digital Pain Scale-Facial expression Scale (NRS-FRS), Chinese version of Self-rating scale of mental fatigue (MFS), and The Chinese version of the multidimensional fatigue directory (MFI-20). Among them, the most evaluated scale was FSS with nine entries, while FSS-7 had two fewer entries with seven items. The NRS-FRS does not explicitly mention the scale dimensions, and FSS, FSS-7, and FACIT-F are all one-dimensional scales, while the rest are multidimensional scales. The scoring methods used for each item included Likert 4 scoring, Likert 5 scoring, and Likert 7 scoring; Likert 5 scoring was the most commonly used item scoring method in the included studies. The interval between the two measurements of the scale ranged from 2 days to 2 months. The basic characteristics of the included PSF correlation measurement scales are detailed in Table 1.

**Table 1:** The basic characteristics of the included PSF correlation measurement scales.

The first author	The year of publication	Country	Scale name	Sample size	Number of scale entries/scale dimension	Scale dimension	Scoring method used for each entry	Time to scale completion (min)	Retest time
Chun-wei Wu (14)	2007	China	FSS	214	9/1	Fatigue consequences	Grade likert 7	NR	NR
Nadarajah M et al. (15)	2017	Malaysia	FSS	100	9/1	Fatigue consequences	Grade likert 7	NR	1 Week
Ozyemisci-Taskiran O et al. (5)	2019	Turkey	FSS	98	9/1	Fatigue consequences	Grade likert 7	NR	1 Week
Abdulla FA et al. (16)	2019	Saudi Arabia	FSS	217	9/1	Fatigue consequences	Grade likert 7	NR	1 Week
Lerdal A et al. (17)	2011	Norway	FSS-7	119	7/1	Fatigue consequences	Grade likert 7	NR	NR
Chun-wei Wu (14)	2007	China	FIS	214	40/3	Physical, cognitive, and social	Grade likert 5	NR	NR
Saneii S H et al. (6)	2020	Iran	FIS	280	40/3	Physical, cognitive, and social	Grade likert 5	10 ~ 20	1 Week
Batur EB et al. (18)	2021	Turkey	FIS	82	40/3	Physical, cognitive, and social	Grade likert 5	NR	1 Week
Smith OR et al. (19)	2008	Holland	FAS	377	10/2	Physical fatigue, mental fatigue	Grade likert 5	NR	Two months
Bråndal A et al. (20)	2016	Sweden	FAS	72	10/2	Physical fatigue, mental fatigue	Grade likert 5	NR	NR
Ho LYW et al. (7)	2021	China	FAS	112	10/2	Physical fatigue, mental fatigue	Grade likert 5	NR	7.22 ± 0.51 days
Chen Hongmei et al. (21)	2020	China	NFI-Stroke	370	12/2	Body, cognition	Grade likert 4	NR	2 Weeks
Taasen I et al. (22)	2020	Norway	NFI-Stroke	66	12/2	Body, cognition	Grade likert 4	NR	2 Days to 1 week
Ho LY et al. (23)	2021	China	NFI-Stroke	177	12/2	Body, cognition	Grade likert 4	NR	7 ~ 10 Days
Chun-wei Wu (14)	2007	China	CIS	214	20/4	Subjective fatigue, attention, motivation, and physical strength	Grade likert 7	NR	NR
Butt Z et al. (24)	2013	America	FACIT-F	399	13/1	NR	Grade likert 5	NR	NR
Chuang LL et al. (25)	2015	China	NRS-FRS	106	NR	NR	NR	NR	1 Week
Liu Xiaoling et al. (26)	2018	China	MFS	295	15/4	Increased sensitivity, fatigue perception, cognitive fatigue, and altered sleep	Grade likert 4	NR	1 Week
Chen Yiting et al. (27)	2022	China	MFI-20	374	20/6	Overall fatigue, attention fatigue, physical fatigue, mental fatigue, reduced activity, and decreased power	Grade likert 5	NR	NR

FSS, Fatigue Severity Scale; FSS-7, Fatigues Severity Scale (7 items); FIS, Fatigue Impact Scale; FAS, Fatigue Assessment Scale; NFI-Stroke, Stroke nerve fatigue refers to Quantity Scale; CIS, Personal Fatigue Strength Questionnaire; FACIT-F, Chronic disease treatment Functional Assessment-Fatigue Scale; NRS-FRS, Digital pain grading-facial expression Scale; MFS, Chinese mental fatigue self-rating scale; MFI-20, multidimensional fatigue scale; and NR, not mentioned.

### 3.3 Methodological quality and measurement attributes quality evaluation results

The 17 included papers (5–7, 14–27) evaluated the content validity, structural validity, internal consistency, hypothesis testing, stability, cross-cultural validity, or measurement invariance of the scales in terms of methodological quality and quality of measurement attributes. (1) Content validity: eight studies (6, 7, 14, 20–23, 26) completed the evaluation of the scale content validity by consulting with experts. However, due to the insufficient description of the evaluation methods and processes adopted by the experts, the methodology quality assessment is “fuzzy” and the content validity is “uncertain.” Five studies (6, 7, 21, 23, 26) asked about patients’ understanding of the content of the scale, and two studies (6, 7) conducted face-to-face interviews with the patients and adjusted the content of the scale based on the interview results, but the methodology quality was assessed as “good” and “good” while the content validity was “sufficient”; the other three studies (21, 23, 26) did not perform qualitative analysis, the methodology quality and content validity corresponded to “fuzzy” and “uncertain.” (2) Structural validity: eight studies (5, 7, 14, 16, 21, 23, 26, 27) reported the test results of the structural validity of the scale. Among them, four studies (14, 16, 21, 27) used only the exploratory factor analysis to evaluate the structural validity of the scale, and the methodological quality was “good”; four studies (5, 7, 23, 26) performed confirmatory factor analysis, one study (5) was “good” due to insufficient sample size, and the other three studies (7, 23, 26) was “very good.” Two studies (23, 26) also reported the comparative fit coefficient of the scale (0.97), thus its construct validity was “sufficient.” (3) Internal consistency: 16 studies (5–7, 14–24, 26, 27) evaluated the internal consistency of the scale; of which, 8 studies (6, 15, 17–20, 22, 24) did not test the structural validity of the scale, the methodological quality corresponds to “fuzzy,” another 8 studies (5, 7, 14, 16, 21, 23, 26, 27) in addition to containing the test results of structural validity, also includes the Cronbach’s α coefficient of each dimension of the scale, thus the methodological quality is “very good.” In one study (23), Cronbach’s α coefficient is <0.7, its internal consistency corresponds to “inadequate.” (4) Hypothesis testing: The methodological quality of 11 studies (5, 7, 15, 16, 18, 20, 21, 23, 25–27) was “good”; 2 studies (6, 17) failed measurement properties or statistical analysis methods. A total of 10 studies (5–7, 15, 17, 18, 21, 23, 26, 27) did not make the hypothesis test as “uncertain,” and the (16, 20, 25) was “sufficient” in the remaining three studies. (5) Stability: 10 studies (5–7, 15, 16, 18, 20, 23, 25, 26) assessed the stability of the scale by test-retest reliability, of which 5 studies (7, 16, 18, 20, 23) were “fuzzy” in terms of methodological quality as they did not specify the measurement situation or retest time. In total, 10 studies (5–7, 15, 16, 18, 20, 23, 25, 26) all had a within-group correlation coefficient of >0.7, thus their stability was “sufficient.” (6) Cross-cultural validity or measurement invariance: only one study (17) conducted differential item functioning (DIF) analysis and the results showed that there is no DIF entry in FSS-7, thus the methodological quality is “very good,” and cross-cultural validity or measurement invariance is “sufficient.” The results of the methodological quality and measurement attribute quality assessment of the PSF measurement scale are shown in Tables 2, 3.

**Table 2:** The evaluation of content validity, structural validity, and internal consistency of PSF measurement scales.

The first author	Scale name	Content validity			Structure validity		Internal consistency	
		Relativity	Comprehensive-ness	Understanding	Index	Evaluation results	Cronbach’s α coefficient	Evaluation results
Chun-wei Wu (14)	FSS	D^a^/?	D^a^/?	NR	EFA:1 factor	A/?	0.93	V/+
Nadarajah M et al. (15)	FSS	NR	NR	NR	NR	NR	0.93	D/?
Ozyemisci-Taskiran O et al. (5)	FSS	NR	NR	NR	CFA:1 factor	A/?	0.93	V/+
Abdulla FA et al. (16)	FSS	NR	NR	NR	EFA:1 factor	A/?	0.93	V/+
Lerdal A et al. (17)	FSS-7	NR	NR	NR	NR	NR	0.87	D/?
Chun-wei Wu (14)	FIS	D^a^/?	D^a^/?	NR	EFA:6 factors	A/?	0.92 ~ 0.94	V/+
Saneii S H et al. (6)	FIS	D^a^/?	D^a^/?	A^b^/+	NR	NR	0.87 ~ 0.95	D/?
Batur EB et al. (18)	FIS	NR	NR	NR	NR	NR	0.80 ~ 0.95	D/?
Smith OR et al. (19)	FAS	NR	NR	NR	NR	NR	0.77	D/?
Bråndal A et al. (20)	FAS	D^a^/?	D^a^/?	NR	NR	NR	0.82	D/?
Ho LYW et al. (7)	FAS	D^a^/?	D^a^/?	A^b^/+	CFA:2 factors	V/+	0.71 ~ 0.78	V/+
Chen Hongmei et al. (21)	NFI-Stroke	D^a^/?	D^a^/?	D^b^/?	EFA:2 factors	A/?	0.80 ~ 0.91	V/+
Taasen I et al. (22)	NFI-Stroke	D^a^/?	D^a^/?	NR	NR	NR	0.74 ~ 0.89	D/?
Ho LY et al. (23)	NFI-Stroke	D^a^/?	D^a^/?	D^b^/?	CFA and EFA:2 factors CFI = 0.97	V/+	0.69 ~ 0.87	V/−
Chun-wei Wu (14)	CIS	D^a^/?	D^a^/?	NR	EFA:4 factors	A/?	0.76 ~ 0.93	V/+
Butt Z et al. (24)	FACIT-F	NR	NR	NR	NR	NR	0.91	D/?
Chuang LL et al. (25)	NRS-FRS	NR	NR	NR	NR	NR	NR	NR
Liu Xiaoling et al. (26)	MFS	D^a^/?	D^a^/?	D^b^/?	CFA and EFA:4 factors CFI = 0.97	V/+	0.92 ~ 0.96	V/+
Chen Yiting et al. (27)	MFI-20	NR	NR	NR	EFA:6 factors	A/?	0.71 ~ 0.86	V/+

a Ask experts.

b Ask patients.

NR, not reported; CFA, confirmatory factor analysis; EFA, exploratory factor analysis; CFI, comparative fit index; ICC, within-group correlation coefficient; DIF, differential item functioning; +, sufficient; −, inadequate;?, unsure; V, very good; A, adequate; D, doubtful; and I, inadequate.

**Table 3:** The evaluation of hypothesis testing, stability, and cross-cultural validity or measurement invariance of PSF measurement scales.

The first author	Scale name	Hypothesis testing		Stability		Cross-cultural validity or measurement invariance	
		Index	Evaluation results	ICC	Evaluation results	Index	Evaluation results
Chun-wei Wu (14)	FSS	NR	NR	NR	NR	NR	NR
Nadarajah M et al. (15)	FSS	2 comparison scales	V/?	0.93	V/+	NR	NR
Ozyemisci-Taskiran O et al. (5)	FSS	3 comparison scales	V/?	0.74	V/+	NR	NR
Abdulla FA et al. (16)	FSS	3 comparison scales	V/+	0.92	D/+	NR	NR
Lerdal A et al. (17)	FSS-7	2 comparison scales	A/?	NR	NR	DIF	V/+
Chun-wei Wu (14)	FIS	NR	NR	NR	NR	NR	NR
Saneii S H et al. (6)	FIS	2 comparison scales	A/?	0.99	V/+	NR	NR
Batur EB et al. (18)	FIS	3 comparison scales	V/?	0.83	D/+	NR	NR
Smith OR et al. (19)	FAS	NR	NR	NR	NR	NR	NR
Bråndal A et al. (20)	FAS	2 comparison scales	V/+	0.73	D/+	NR	NR
Ho LYW et al. (7)	FAS	4 comparison scales	V/?	0.92	D/+	NR	NR
Chen Hongmei et al. (21)	NFI-Stroke	1 comparison scale	V/?	NR	NR	NR	NR
Taasen I et al. (22)	NFI-Stroke	NR	NR	NR	NR	NR	NR
Ho LY et al. (23)	NFI-Stroke	4 comparison scales	V/?	0.93	D/+	NR	NR
Chun-wei Wu (14)	CIS	NR	NR	NR	NR	NR	NR
Butt Z et al. (24)	FACIT-F	NR	NR	NR	NR	NR	NR
Chuang LL et al. (25)	NRS-FRS	1 comparison scale	V/+	0.95	V/+	NR	NR
Liu Xiaoling et al. (26)	MFS	1 comparison scale	V/?	0.85	V/+	NR	NR
Chen Yiting et al. (27)	MFI-20	1 comparison scale	V/?	NR	NR	NR	NR

Same as “note” at the bottom of Table 2.

### 3.4 Measurement attribute synthesis results and recommendations

For content validity, FSS-7, FACIT-F, NRS-FRS, and MFI-20 were “not mentioned” and the remaining scales were “uncertain.” The construct validity of the MFS was “sufficient,” the FSS-7, FACIT-F, and NRS-FRS were “not mentioned,” and the remaining scales were “uncertain.” The internal consistency of the NRS-FRS was “not mentioned,” the FSS and MFS were “sufficient,” and the internal consistency of the remaining scales was”uncertain.” The hypothesis tests for CIS and FACIT-F were “not mentioned,” NRS-FRS “sufficient,” and the remaining scales were “uncertain.” For stability, FSS-7, CIS, FACIT-F, and MFI-20 are “not mentioned” and the remaining scales are “sufficient.” Cross-cultural validity or measurement invariance of the FSS-7 was “full,” and the rest of the scales were “not mentioned.” Due to the risk of bias, the quality of evidence for the measurement attributes included in this study is mainly “medium” or “low,” and the recommendation grade is “B.” The synthetic results and recommendations of the PSF measurement scale are shown in Table 4.

**Table 4:** The synthetic results and recommendations of the PSF measurement scale.

Scale name	Content validity		Structure validity		Internal consistency		Hypothesis test		Stability		Cross-cultural validity or measurement invariance		Recommended grade
	Overall rating	Quality of evidence	Overall rating	Quality of evidence	Overall rating	Quality of evidence	Overall rating	Quality of evidence	Overall rating	Quality of evidence	Overall rating	Quality of evidence	
FSS	?	Middle	?	Middle	+	High	?	Middle	+	Middle	NR	NR	B
FSS-7	NR	NR	NR	NR	?	Middle	?	Middle	NR	NR	+	High	B
FIS	?	Middle	?	Low	?	Middle	?	Middle	+	Middle	NR	NR	B
FAS	?	Middle	?	Middle	?	Middle	?	High	+	Middle	NR	NR	B
NFI-Stroke	?	Middle	?	Middle	?	Middle	?	Middle	+	Middle	NR	NR	B
CIS	?	Middle	?	Low	?	Middle	NR	NR	NR	NR	NR	NR	B
FACIT-F	NR	NR	NR	NR	?	Low	NR	NR	NR	NR	NR	NR	B
NRS-FRS	NR	NR	NR	NR	NR	NR	+	Middle	+	Middle	NR	NR	B
MFS	?	Middle	+	High	+	High	?	Middle	+	Middle	NR	NR	B
MFI-20	NR	NR	?	Low	?	Middle	?	Middle	NR	NR	NR	NR	B

+, sufficient; −, inadequate;?, unsure; and NR, not mentioned.

## 4 Discussion

This study included 17 studies involving 10 PSF measurement scales. Although there are many scales available to measure PSF, they have different priorities for evaluating PSF, and the quality of their measurement properties is uneven, thus the corresponding test methods also have some problems. In this study, the existing issues in the included scale were analyzed and summarized from both scale validity and reliability, and relevant recommendations combined with the scale dimension were made, aiming to provide some theoretical basis for the selection, verification, or development of PSF-related measurement scales in the future.

Content validity, as the most important measurement attribute of a scale, can directly affect the level of evidence scale. However, most of the included studies did not explicitly mention the evaluation of the content validity of the scale, and only a small number of studies focused on the understanding of the scale. Based on this, in future research involving the development or verify the validity of the scale, in addition to the evaluation of scale validity, the researchers should also try to implement face-to-face interviews with the subjects, so as to intuitively understand the patient’s understanding of the scale, and according to the results of the scale content, in order to improve the agreement between the content of the scale and the tested constructs (28). Most of the included studies did not assess the structural validity of the scales. Furthermore, future studies need to present hypotheses between comparative scales before hypothesis testing; cross-cultural validity or measurement invariance should be evaluated. The scale reliability involved in this study included internal consistency and stability. It should be noted that when evaluating the internal consistency of multidimensional scales, the Cronbach’s α coefficient of the total scale and each subscale should be calculated simultaneously to clarify the reliability of each dimension of the scale. The stability of the scales included in this study was responded to by test–retest reliability. At present, researchers choose the retest time based on the hospitalization time of patients or from their own experience. There is no unified standard, which will affect the retest reliability of the scale to some extent. Therefore, future relevant studies should not only clarify the time interval between the two measurements but also clarify the basis for the selection of the retest time.

None of the scales included in this study evaluated responsiveness, measurement error, and criterion validity. Because the causal mechanism and specific features of PSF are still largely unknown (29), there is no golden standard for measuring PSF, thus it is impossible to evaluate the validity of PSF-related measurement scales. The FSS-7 included in this study was obtained after deleting two entries from the original FSS, but the study did not compare it with the original FSS to evaluate standard validity. Although Lenaert et al. (30) highlighted that there was a weak to moderate and strong correlation between the fatigue experience of patients with PSF and the FSS and FSS-7 scales, it also did not mention the content related to validity. Based on this, when the new scale is developed based on the original scale in the future, the new scale can be compared with the original scale, so as to complete the evaluation of the validity standard.

This study showed that FSS was the most evaluated scale, followed by FIS, FAS, and NFI-stroke, and none of these scales were evaluated for cross-cultural validity or measurement invariance. Although FSS-7 evaluated cross-cultural validity or measurement invariance, only one included study evaluated CIS, FACIT-F, NRS-FRS, MFS, and MFI-20, and more studies are needed to test whether the above scale is clinically applicable in the future. FSS has 9 items, and the evidence quality level of content validity and internal consistency corresponds to “medium” and “high,” respectively, which are also widely used in clinical practice. This is consistent with the findings of Kjeverud et al. (8). More research is needed in the future to clarify and harmonize the measurement tools for PSF. Therefore, this study temporarily recommends FSS for PSF measurement, but more tests still need to be conducted on its relevant measurement attributes, especially the cross-cultural validity or measurement invariance. In addition, the FSS is a unidimensional scale that can only assess somatic fatigue. For a multidimensional PSF assessment, perhaps FIS, FAS, and NFI-stroke should be considered. However, the above measurement scale should also be tested with more relevant measurement properties to verify its clinical applicability. This study also has some limitations as follows: ① only Chinese and English literature are included, there may be language bias; ② PSF scale has only one study included in the evaluation, which may affect the results of this study to some extent; ③ some scales have not been studied with large samples, thus the results of this study need to be interpreted carefully.

## 5 Conclusion

In summary, this study temporarily recommended FSS to measure PSF. To assess the PSF more comprehensively, the use of FIS, FAS, and NFI-stroke should be considered. All 10 PSF measurement scales involved in this study need to be studied to verify their validity. In future, when selecting, validating, or developing PSF-related measurement scales, the relevant assessment problems of the inclusion scales mentioned in this study should be avoided as far as possible, in order to get more high-quality evidence support and more scientific and standardized measurement tools.
